# Construction and validation of educational technology in the form of a booklet to guide the elderly in post-burn care

**DOI:** 10.15649/cuidarte.5229

**Published:** 2026-08-28

**Authors:** Marcelo Anderson Cavalcante Monteiro, Odézio Damasceno Brito, Jamily Soares Damasceno Silva, Ana Clécia Silva Monteiro, Leticia Coelho de Souza, Anne Fayma Lopes Chaves, Livia Moreira Barros, Natasha Marques Frota

**Affiliations:** 1 Universidade da Integração Internacional da Lusofonia Afro-Brasileira, Redenção, Brazil. E-mail: marcelocavalcanteenfermeiro@gmail.com Universidade da Integração Internacional da Lusofonia Afro-Brasileira Redenção Brazil marcelocavalcanteenfermeiro@gmail.com; 2 Universidade da Integração Internacional da Lusofonia Afro-Brasileira, Redenção, Brazil. E-mail: odeziod@gmail.com Universidade da Integração Internacional da Lusofonia Afro-Brasileira Redenção Brazil odeziod@gmail.com; 3 Universidade da Integração Internacional da Lusofonia Afro-Brasileira, Redenção, Brazil. E-mail: jamilysoares@hotmail.com Universidade da Integração Internacional da Lusofonia Afro-Brasileira Redenção Brazil jamilysoares@hotmail.com; 4 Universidade da Integração Internacional da Lusofonia Afro-Brasileira, Redenção, Brazil. E-mail: clecia.monteiro_26@hotmail.com Universidade da Integração Internacional da Lusofonia Afro-Brasileira Redenção Brazil clecia.monteiro_26@hotmail.com; 5 Universidade da Integração Internacional da Lusofonia Afro-Brasileira, Redenção, Brazil. E-mail: coelholeticia595@gmail.com Universidade da Integração Internacional da Lusofonia Afro-Brasileira Redenção Brazil coelholeticia595@gmail.com; 6 Universidade da Integração Internacional da Lusofonia Afro-Brasileira, Redenção, Brazil. E-mail: annefayma@unilab.edu.br Universidade da Integração Internacional da Lusofonia Afro-Brasileira Redenção Brazil annefayma@unilab.edu.br; 7 Universidade da Integração Internacional da Lusofonia Afro-Brasileira, Redenção, Brazil. E-mail: livia@unilab.edu.br Universidade da Integração Internacional da Lusofonia Afro-Brasileira Redenção Brazil livia@unilab.edu.br; 8 Universidade da Integração Internacional da Lusofonia Afro-Brasileira, Redenção, Brazil. E-mail: natasha@unilab.edu.br Universidade da Integração Internacional da Lusofonia Afro-Brasileira Redenção Brazil natasha@unilab.edu.br

**Keywords:** Aged, Burns, Validation Studies, Patient Education Handout, Patient Education, Anciano, Quemaduras, Estudios de Validación, Folleto Informativo para Pacientes, Educación del Paciente, Idoso, Queimaduras, Estudos de Validação, Prospecto para Educação de Pacientes, Educação do Paciente

## Abstract

**Introduction:**

The aging population necessitates increased healthcare focus, as the incidence of hospitalizations and complications, especially those related to burns, rises. Consequently, it is urgent to educate the elderly on self-care to prevent severe complications.

**Objective:**

To develop and validate an educational booklet to guide elderly individuals in post-burn care at home.

**Materials and Methods:**

This was a methodological study conducted in four stages: literature review, booklet development, expert validation and target audience evaluation. Data was analyzed using descriptive statistics and the Content Validity Index.

**Results:**

A total of 23 experts with experience in elderly health, education and technology and 23 elderly participants followed by a burn treatment center participated. The final booklet, titled “Protecting Your Skin: Guidelines for Post-Burn Care”, includes 51 pages and covers topics such as burn classification, initial care, accident prevention, and useful contacts. The content validity index was 1.0 among experts and 0.92 among older adults, indicating excellent acceptance and understanding of the content.

**Discussion:**

The booklet addresses post-discharge of health education needs of older adults with burns, standing out for the target audience’s comprehension of educational technology, accessible language and evidence-based content.

**Conclusion:**

The educational technology in the form of a booklet demonstrated adequate validation and a high level of understanding among older adults, showing potential for incorporation into nursing practice as an educational resource, promoting self-care and the safety of older adults in post-hospital discharge care.

## Resumen

**Introducción:**

El envejecimiento de la población requiere mayor atención sanitaria dado el aumento de las hospitalizaciones y las complicaciones, especialmente en casos de quemaduras. Por lo tanto, resulta urgente educar a la población sobre el autocuidado para prevenir complicaciones.

**Objetivo:**

Construir y validar un folleto educativo para guiar el conocimiento sobre el cuidado posterior a quemaduras en el hogar.

**Materiales y métodos:**

Estudio metodológico realizado en cuatro etapas: revisión bibliográfica, recopilación de datos, validación por expertos y evaluación con el público. Participaron 23 expertos con experiencia en salud, educación y tecnologías, junto con 23 expertos de un centro especializado en quemaduras. Los datos se analizaron mediante estadística descriptiva y el cálculo del Índice de Validez de Contenido.

**Resultados:**

El folleto final, titulado "Protegiendo su piel: Guía para el cuidado posterior a quemaduras", consta de 51 páginas y aborda temas como la clasificación de quemaduras, primeros auxilios, prevención de accidentes y contactos útiles. El índice de validez fue de 1,0 entre los expertos y de 0,92 entre los usuarios, lo que indica una excelente aceptación y comprensión del contenido.

**Discusión:**

El folleto aborda las necesidades de educación sanitaria de las víctimas de quemaduras tras el alta hospitalaria, haciendo hincapié en la comprensibilidad de la tecnología educativa para el público general, el lenguaje accesible y el contenido basado en la evidencia.

**Conclusiones:**

La tecnología educativa sobre el folleto muestra una validación adecuada y un alto grado de comprensión por parte de los pacientes, lo que demuestra su potencial para incorporarse a la práctica clínica como recurso educativo, promoviendo el autocuidado y la seguridad de las personas sanas tras el alta hospitalaria.

## Resumo

**Introdução:**

O envelhecimento populacional requer maior atenção à saúde. pois a incidência de internações e complicações aumenta, especialmente no caso de queimaduras. Assim, torna-se urgente educar a população idosa sobre o autocuidado para prevenir complicações.

**Objetivo:**

Construir e validar uma cartilha educativa para orientar idosos sobre cuidados após queimaduras em contexto domiciliar.

**Materiais e métodos:**

Estudo metodológico realizado em quatro etapas: levantamento bibliográfico, construção da cartilha, validação por especialistas e avaliação com o público-alvo. Os dados foram analisados com estatísticas descritivas e cálculo do Índice de Validade de Conteúdo.

**Resultados:**

Participaram 23 especialistas com experiência em saúde do idoso, educação e tecnologias, e 23 idosos acompanhados por um centro especializado em queimaduras. A cartilha final, intitulada“Protegendo sua pele: Orientação para cuidados após queimaduras”, possui 51 páginas e aborda temas como classificação das queimaduras, primeiros cuidados, prevenção de acidentes e contatos úteis. O índice de validade foi 1,0 entre os especialistas e 0,92 entre os idosos, indicando excelente aceitação e compreensão do conteúdo.

**Discussão:**

A cartilha responde às necessidades de educação em saúde no pós-alta de idosos queimados, destacando-se pela compreensão da tecnologia educativa pelo público-alvo, linguagem acessível e conteúdo baseado em evidências.

**Conclusões:**

A tecnologia educativa do tipo cartilha apresentou validação adequada e elevado grau de compreensão pelos idosos, demonstrando potencial para ser incorporada na prática de enfermagem como recurso educativo, promovendo o autocuidado e a segurança da pessoa idosa no pós-alta hospitalar.

## Introduction

In recent decades, a significant demographic shift has been observed in the global population and social aging has become an increasingly present reality. It is estimated that, by 2050, the world's elderly population will have doubled, reaching 2.1 billion^1^. This accelerated growth of the elderly population brings with it significant challenges for health systems, social security and public policies aimed at the well-being of this age group^2^.

This increase demands more effective healthcare, especially due to the high rate of hospitalizations and the risk of complications, such as burns^3^,^4^. Between 2010 and 2019, the mortality rate from burns in the elderly exceeded that of the general population, particularly in the North Region, where there was an increase of 1,046.6% (from 1.35% to 15.48%)^3^. These numbers highlight the need to raise awareness among the elderly population about self-care after burns to avoid serious complications and deaths, especially in regions with difficult access to specialized care services.

Furthermore, there are cases of people who have already suffered some type of burn returning to the health service with the same type of injury^5^. This highlights the importance of health education for the elderly and their families, focusing on burn healing, continuity of care and prevention of new injuries^6^,^7^.

For this reason, the use of educational technologies has shown potential to promote the proper management of burns and reduce inconsistencies in information^8^. Thus, the development of tools aimed at this audience not only promotes healing and the prevention of new injuries, but also meets the global need to promote evidence-based educational strategies that minimize the health burden and socioeconomic impacts of burns in different contexts^9^.

A study conducted in New York showed that the distribution of educational kits made it possible to reduce burn injuries by 67% among elderly people^10^. Other educational technologies have been developed for healthcare professionals and family members of elderly people^11^,^12^. Thus, the production of booklets is the preferred technology for elderly people and the content being geared towards the patient learning about their health condition can promote autonomy and self-care^13^.

Given the above, the objective of this study was to construct and validate educational technology, in the form of a booklet, to guide elderly people in the care of burns after hospital discharge.

## Materials and Methods

This methodological study, developed in four stages adapted from Echer^14^, involved: a literature review; the creation of a booklet on elderly care; validation of the booklet with experts; and validation with the target audience. The research took place between July and September 2024 at the Burn Treatment Center (CTQ) of the reference hospital for burn care in the state of Ceará.

The literature review was conducted using an integrative review that employed the PICO strategy to formulate the research question: "What nursing care is provided to burn victims?". The databases chosen were: Latin American and Caribbean Health Sciences Database (LILACS), Nursing Database (BDENF), Spanish Bibliographic Index in Health Sciences (IBECS), Scientific Electronic Library Online (Scielo), Pan American Health Organization (PAHO), PubMed Central and Google Scholar.

For the selection of studies, a screening was conducted through the evaluation of titles and abstracts by two different researchers, using a free web-based review program called Rayyan Qatar Computing. Research The Institute (Rayyan QCRI)^15^, due to its potential to add efficiency and ease to the article screening process. Next, the articles that met the inclusion criteria were read in full. These criteria were: original research that answered the guiding question, available electronically in full, in any language and without a time restriction. Exclusion criteria included theses, dissertations, letters to the editor, opinion pieces and duplicate studies.

Based on the results of the integrative review, the development of the booklet began in collaboration with a graphic designer to assist in the development of the illustrations and the layout of the educational material, which were adapted for colloquial language.

For validation, the methodological framework used was based on the parameters proposed by Alexandre and Coluci^16^ for content validation studies in health, considering the domains of clarity, pertinence, relevance and presentation, with a Content Validity Index (CVI) calculated as ≥ 0.80.

The research involved professionals from the Burn Treatment Center – CTQ. prioritizing experts with nursing training and experience in elderly health, technologies and care for burn victims. To this end initial contact was made through communication mechanisms such as email, inviting them to participate in the validation. It is worth noting that, to participate in the research, the criterion adopted was that the content expert meets a minimum of five points^17^.

Sample size calculation was performed using the formula n=Za².P.(1− P)/e², applied to both the expert group and the target audience. The parameters used were a 95% confidence level, an expected proportion of 85% and a 15% margin of error. For the expert group, the sample was adjusted to 24 participants, with an additional 10% to mitigate the absence of responses. The selection of experts was carried out through snowball sampling, where the participants themselves indicate other experts, an ideal method for finding professionals in specific niches^18^.

An invitation letter was sent to the email addresses of those who met the established criteria. After acceptance, the Informed Consent Form (ICF), the first version of the booklet and the adapted evaluation instrument^19^, were made available via Google Forms. The experts evaluated the material and suggested improvements, which were analyzed and incorporated when pertinent.

It should be noted that experts were given 15 days to respond to the evaluation of the booklet. Participants who did not meet this deadline were contacted again, granting an additional 15 days, totaling 30 days for feedback. Two days before the deadline, an email was sent informing participants of the remaining time for feedback. Participants who did not submit the material by the stipulated deadline were excluded from the research.

The inclusion criteria were: age ≥60 years, history of burns under outpatient follow-up at the service, care provided at home, literacy in Portuguese with the ability to read and understand the material or presence of the primary caregiver responsible for home care willing to participate and clinical stability at the time of assessment. Exclusion criteria included: moderate to severe cognitive impairment that prevented reading/comprehension and uncorrectable visual or auditory impairments that prevented reading.

The screening of elderly participants took place at the hospital during their outpatient follow-up appointments. Two copies of the Informed Consent Form (ICF) were provided, explaining the research and requesting agreement to participate; one copy remained with the participant and the other with the researcher. During data collection, a structured script developed by the author was applied, characterizing the clinical, socioeconomic and demographic profile of the participants, followed by reading of the educational booklet. Afterwards, the booklet was given to the participant to read, with no maximum time limit stipulated for this activity. At this time, the researcher remained close to the participant in case there were any doubts or questions.

After reading, participants were asked to complete the data collection instrument. The instrument was adapted^19^ for this study and assessed 12 items, focusing on the appearance and illustration of the material. Responses were recorded on a five-point Likert scale, from 'strongly disagree' to 'strongly agree'. In addition, the author added the option of writing an additional opinion if the instrument items did not fully address the participant's suggestions for improving the technology.

For content validation, the Content Validity Index (CVI) was used to measure the level of agreement among judges on each item evaluated. A minimum index of 0.80 was considered acceptable, with values above 0.90 being preferred. The CVI was calculated by summing the judges' agreement responses, coded as 0 (inadequate), 1 (partially adequate) and 2 (adequate) and divided by the total number of judges^16^,^20^,^21^.

For data tabulation and analysis, Microsoft Excel for Windows and Epi Info™ software, version 7.0 for Windows, were used, employing the binomial test to verify the significance of agreement between evaluators. The collected data are available in open access on the Mendeley Data^22^ platform, ensuring transparency and reproducibility of the results.

The study was approved by the Research Ethics Committee of the Dr. José Frota Institute, under opinion number 6,602,953 and all participants signed the Informed Consent Form.

## Results

The initial version of the booklet was validated by expert teachers and healthcare professionals with experience in the areas of elderly health, public health, or technology. Twenty-three content experts participated in the validation study and their characterization data are presented in Table 1.

**Table 1:** Characterization of expert judges. Ceará, Brazil, 2025. n= (23)

Variables	% (n)
Age, Mean ± SD	50.00 ± 10.13
Age Range	
31 to 39 years old	21.73 (5)
40 to 69 years old	73.91 (17)
70 years or older	4.34 (1)
Sex	
Female	69.55 (16)
Male	30.42 (7)
Training	
Nursing	69.55 (16)
Physiotherapy	8.69 (2)
Medicine	8.69 (2)
Nutrition	4.34 (1)
Psychology	4.34 (1)
Occupational Therapy	4.34 (1)
Training Time (in years), Mean ± SD	23.87 ± 10.54
Time working in the field (in years), Mean ± SD	17.26 ± 10.64
Titration	
Master's degree	34.78 (8)
PhD	60.86 (14)
Postdoctoral studies	4.34 (1)
Area of Expertise	
Assistance	34.77 (8)
Teaching	13.04 (3)
Assistance and Teaching	43.47 (10)
Teaching and Technologies	8.78 (2)

SD: Standard deviation; n: number

For the evaluation of the technology with the target audience, 23 elderly individuals participated in the research. To avoid ties in the evaluated items, these were elderly burn victims who were being monitored by the Burn Treatment Center (CTQ). Table 2 presents the characterization data of the elderly individuals participating.

**Table 2:** Characterization of the target audience of the booklet. Ceará, Brazil, 2025. n= (23)

Variables	% (n)
Age Range	
Age, Mean ± SD	65.22 ± 4.59
60 to 69 years old	78.24 (18)
70 years or older	21.73 (5)
Sex	
Female	43.47 (10)
Male	56.51 (13)
Color/Race	
Brown	17.38 (4)
White	52.16 (12)
Black	26.08 (6)
Not informed	4.34 (1)
Marital status	
Single	13.04 (3)
Married	52.16 (12)
Divorced	17.38 (4)
Widower	21.73 (5)
Occupation	
Teacher	8.69 (2)
Cook	4.34 (1)
Dressmaker	4.34 (1)
Driver	4.34 (1)
Housekeeper	8.69 (2)
Farmer/Market Vendor	30.42 (7)
Self-employed	8.69 (2)
Retiree	21.73 (5)
Businessperson	4.34 (1)
Seller	4.34 (1)
Family Income	
1 minimum wage	17.38 (4)
2 minimum wages	82.59 (19)
kind of live	
Urban Zone	69.55 (16)
Rural Area	30.42 (7)
Type of Residence	
Home	86.94 (20)
Apartment	13.04 (3)

SD: Standard deviation; n: number

The final version of the booklet “Protecting Your Skin: Guidance for Aftercare” has 51 pages, including the cover, back cover, technical data sheet, acknowledgments and table of contents. The booklet is structured around eight topics: 1) What are burns; 2) Classification of burns; 3) First aid for burns; 4) Necessary guidelines and care; 5) Tips on how to avoid domestic accidents; 6) Myths and truths about burns; 7) Important contacts; and 8) Notes on follow-up appointments.

The primary objective of this booklet was to gather educational information aimed at elderly individuals who have suffered burns, undergone hospitalization and need to return home.

The validation phase of the educational material involved the analysis of the content by 23 experts, with the aim of forming a multidisciplinary team, which included: nurses, physiotherapists, doctors, nutritionists, psychologists and occupational therapists.

Most of the experts were nurses (69.60%). They were female (69.60%) and had experience in both care and teaching (43.52%), aged between 40 and 69 years (73.90%), with an average age of 50.00 ± 10.13 years. The judges had an average of 23.87 ± 10.54 years of professional experience. The profile of these professionals is notable for their high academic qualifications, with 60.91% holding a doctorate and for their extensive professional experience, with an average of 17.26 years.

In the validation process through the calculation of the overall IVC (Content Validity Index), the average score was one point. Therefore, according to the IVC cutoff point recommended in the literature, the prepared booklet is considered validated in terms of the content presented. Table 3 shows the items evaluated in this stage).

**Table 3:** Distribution of expert agreement regarding the content validation of the educational booklet on burns. Ceará, Brazil, 2025. n= (23)

Items	%(n)	IVC	p-value
Objectives			
1. Addresses the proposed theme.	100 (23)	1	1.000
2. Suitable for the teaching-learning process.	100 (23)	1	1.000
3. Clarify any possible doubts about the topic discussed.	100 (23)	1	1.000
4. It provides food for thought on the topic.	100 (23)	1	1.000
5. Encourages behavioral change.	100 (23)	1	1.000
Structure and presentation	100 (23)		
6. Language appropriate to the target audience.	100 (23)	1	1.000
7. Language appropriate to educational material.	100 (23)	1	1.000
8. Interactive language.	100 (23)	1	1.000
9. Correct information.	100 (23)	1	1.000
10. Objective information.	100 (23)	1	1.000
11. Clarifying information.	100 (23)	1	1.000
12. Necessary information.	100 (23)	1	1.000
13. Logical sequence of ideas.	100 (23)	1	1.000
14. Current topic.	100 (23)	1	1.000
15. Appropriate text size.	100 (23)	1	1.000
Relevance			
16. Technology stimulates learning.	100 (23)	1	1.000
17. Contributes to knowledge in the area.	100 (23)	1	1.000
18. It sparks interest in the topic.	100 (23)	1	1.000

IVC: Item Content Validity Index; P-value: Binomial Test; n: number

The 18 items evaluated within the three domains were classified as valid. All items received 23 (100%) positive responses. However, despite this, based on the comments and suggestions of the experts, some modifications were adopted to improve educational technology.

Eighteen comments/suggestions were made by the experts. All subjective observations were grouped into the "Structure and Presentation" domain. Twelve suggestions were accepted and six were not, as detailed in Table 4.

**Table 4:** Main observations made by expert judges, Ceará, Brazil. 2025

Domain	Suggestion	Evaluation
Structure and Presentation	Reorganize the order of the topics "Service Routines. Returns. and Special Dressing" to the end of the booklet. Add the need for DT vaccination in some cases. Change the word "NO," as the public may not accept the guidance. Motor rehabilitation was not considered. Cover: restructure, to something more formal. Replacing the "little fire" image with a healthcare professional. Suggestion: put a line like "SENIOR PERSON'S NAME" on the front or back cover. To highlight the importance of the nurse in this care context. Organize the sequence of items more thoroughly. To clarify the different types of soaps. Clarify what is considered good nutrition and what hydration is in the "Important" section. Wear "sun-protective clothing," as not everyone knows what UV protection is. Remove the parentheses (itch), leave the word outside the parentheses. On page 26, move the phrase "This procedure is done with anesthesia" to before the phrase that says to avoid scratching. Add a page at the end of the booklet with the following phrase: " If you have any questions that have not been answered here, ask a registered nurse at the health service." Include photos and images of real burns, along with the page owner. Place the phrase "STAY TUNED" in a way that makes it stand out more; suggestion: put it inside a frame. On page 3, insert the phrase "Learn about some service routines" after "First aid for burns".	Accepted Not Accepted Accepted Not Accepted Accepted Not Accepted Accepted Not Accepted Accepted Accepted Accepted Accepted Accepted Accepted Accepted Not Accepted Not Accepted Accepted

DT: Diphtheria and Tetanus; UV: Ultraviolet radiation

The evaluation phase of the educational booklet with the target audience consisted of an analysis of the visual presentation of the material by 23 elderly individuals. Most reviewers from the target audience were farmers or market vendors (30.41%), male (56.50%), of white race/color (52.23%), aged between 60 and 69 years (78.31%), with a mean age of 65.22 ± 4.59 years. Furthermore, most participants were married (52.23%), with an income of two minimum wages (82.66%) and residents of the urban area of the city (69.60%). Table 5 presents the distribution of agreement among the target audience regarding the educational booklet.

**Table 5:** Distribution of agreement among the target audience regarding the content evaluation of the educational booklet on burns. Ceará, Brazil, 2025. n= (23)

Items	% (n)	IVC	p-value
1. The illustrations are appropriate for the target audience.	100 (23)	1	1.000
2. The illustrations are clear and easy to understand.	95.65 (22)	0.95	0.976
3. The illustrations are relevant for the target audience to understand the content.	86.95 (20)	0.86	0.692
4. The colors in the illustrations are appropriate for the type of material.	91.30 (21)	0.91	0.879
5. The shapes of the illustrations are appropriate for the type of material.	82.60 (19)	0.82	0.460
6. The illustrations depict the daily life of the target audience of the intervention.	86.95 (20)	0.86	0.692
7. The arrangement of the figures is in harmony with the text.	95.65 (22)	0.95	0.976
8. The figures used illustrate the content of the educational material.	95.65 (22)	0.95	0.976
9. The illustrations help in presenting the theme and are in a logical sequence.	100 (23)	1	1.000
10. The illustrations are present in adequate quantity in the educational material.	91.30 (21)	0.91	0.879
11. The illustrations are of appropriate sizes in the educational material.	91.30 (21)	0.91	0.879
12. Illustrations help change the behaviors and attitudes of the target audience.	100 (23)	1	1.000

IVC: Item Content Validity Index; n: number; P-value: Binomial Test

In the target audience validation process, through the calculation of the IVC (Content Validity Index), it presented an average of 0.92 points, being considered validated according to the cutoff point. Finally, the validated guide covers important topics addressing essential guidelines and care for the first few days after a burn and hospital discharge.

## Discussion

The booklet proved to be an educational tool for promoting self-care and preventing further injuries in older adults. The literature highlights that educational interventions are essential to improve adherence to treatment and minimize complications in vulnerable populations, such as older burn victims^23^.

Population aging imposes additional challenges to rehabilitation and functional independence after burns, since factors such as loss of skin elasticity, comorbidities and reduced healing capacity compromise recovery^24^. The developed booklet addressed these issues, providing clear guidelines on mobility, hydration, sun protection and pain management, critical aspects for the recovery and quality of life of these patients^25^-^28^.

Furthermore, the booklet incorporated guidelines for burn prevention, a need documented in the literature, given the high risk of domestic accidents in this population^29^. Adapting the home environment, the proper use of safety equipment and caregiver education are essential measures to minimize these risks. Studies indicate that structured interventions, such as educational materials, contribute significantly to the reduction of adverse events and hospitalizations related to burns in the elderly^30^.

Emotional and social aspects affected, such as feelings of dismemberment, identity disorder and anxiety in relation to the social world, are explored in the booklet, which encourages the promotion of social and leisure activities adapted to the limitations of the elderly, with due attention to signs of depression or anxiety as early as possible^31^,^32^.

A study conducted in a tertiary hospital in India with burn patients evaluated the frequency of depression, anxiety and stress in post-burn patients and their association with self-esteem and social support. The results indicated that 5.3% of participants presented moderate depression, 6.4% moderate anxiety and 8.5% moderate stress. These findings reinforce the idea that interventions can promote self-esteem and social support to reduce symptoms of depression, anxiety and stress in post-burn patients^33^. Accordingly, the booklet developed in this study includes guidelines to encourage social and leisure activities adapted to the limitations of the elderly, with early attention to signs of depression or anxiety, seeking to promote social integration, self-care and mental health in this group.

Validation of the booklet by judges and the target audience reinforced its practical applicability, with high rates of acceptance and comprehension. The overall Content Validity Index (CVI) of 1.0 among experts and 0.92 among the elderly suggests that the booklet meets the criteria of clarity, relevance and clinical applicability, making it a promising resource for health education in this population.

Another study focused on the construction and validation of an educational booklet for the elderly, with a focus on sleep hygiene, used a similar methodology, with validation among specialists and the target audience, also presenting a high CVI of 0.95. Although the topic addressed was different, the results converge with those of this study by highlighting the effectiveness of printed materials as accessible and understandable tools for the elderly population^34^.

Regarding the evaluation with the target audience, another study focused on validating a booklet for burn patients used a different strategy, based on patient reports and evaluation through interviews and focus groups. Although methodologically different, this study also obtained satisfactory results regarding the acceptance and usefulness of the material among patients, reinforcing the importance of educational technologies aimed at the rehabilitation of people affected by burns, including the elderly population^35^.

Evidence from a clinical trial conducted in Iran showed that multimedia education programs. mediated by the WhatsApp application in the post-discharge period, contributed to improving the quality of life of burn patients. These results suggest that educational interventions that integrate informative and rehabilitation components promote the well-being and recovery of individuals^36^. In line with this, the present study highlights that the validated educational booklet can constitute a resource that promotes autonomy for the elderly, assisting in the prevention of complications and strengthening the well-being of this population.

The content of the booklet is presented in simple and accessible language, without scientific terms, using short and direct sentences. The organization into lists and topics facilitates reading, and the inclusion of examples and practical cases illustrates the application of the guidelines in the daily lives of the elderly^37^. The guidelines highlighted in the literature seek to ensure that the booklet is effective and accessible, promoting self-care and the health of the elderly after hospital discharge, aligned with a user-centered approach.

It is known that educational materials aimed at the elderly are more effective when they use simple language, short sentences, organization into lists or topics and the use of illustrations and practical examples.

A quasi-experimental study conducted in rural community centers in Taiwan demonstrated that educational materials developed according to the "EZ to Read" model, with simplified language, short sentences, accessible layout and appropriate visual resources, resulted in significant improvement in the reading comprehension of older adults. These findings show that educational formats adapted to the cognitive and sensory characteristics of the elderly population favor the understanding of information and its application in daily life, since they are designed to facilitate reading for people with low levels of education, cognitive limitations, or who are elderly^38^. In line with this, the booklet validated in the present study was constructed based on similar principles, using clear language, organization into topics and practical examples. This approach reinforces the booklet's potential to promote the autonomy of the elderly in the home management of post-burn care, contributing to the prevention of complications and the promotion of the well-being of this population.

It is believed that the development of the booklet by researchers working in the burn unit of a referral hospital facilitated the construction process, with a focus on the guidelines developed by nurses and the educational needs of the elderly and their families.

The implementation of this educational technology in health services can expand the support offered to elderly burn victims and their caregivers, thus strengthening the continuity of care after discharge. The involvement of nurse educators in this process is essential, ensuring that patients receive adequate information and can correctly apply the guidelines in the home setting^39^.

It is thus highlighted that nurses play an active role in assessing the needs of burn patients, planning individualized training and ensuring their effectiveness. Therefore, this booklet covers important topics to be addressed with hospitalized elderly burn patients and can assist nurses in pre-discharge education, which is essential for adaptation to recovery and the new routine^40^.

Thus, this study contributes to the literature on educational technologies applied to geriatric nursing, offering a validated intervention model that can be incorporated into rehabilitation and burn prevention programs for the elderly. Future studies can explore the effectiveness of the booklet in different contexts and its adaptation to digital platforms, expanding its reach and impact on the care of this population.

Regarding limitations, it is noted that the validation was performed in a single specialized center, which may limit the generalization of the findings, as they belong to a sociocultural context. The researchers' network of contacts, achieved through snowball sampling, may have positively influenced the results and increased the validation rates. Additionally, the absence of longitudinal follow-up made it impossible to assess the impact of the booklet on behavioral change, adherence to guidelines and prevention of new burns after hospital discharge.

## Conclusions

It is concluded that the educational technology of the booklet type achieved its objective by adequately constructing and validating an educational booklet to guide elderly people on post-burn care in a home environment. The booklet, "Protecting Your Skin: Guidance for Post-Burn Care," was developed based on scientific evidence and demonstrated relevance, clarity and suitability through the validation process with experts and the target audience.

Validation confirmed that the material is clear, objective. and functional for its purpose, representing an effective tool for health education. The use of this booklet has the potential to empower the elderly population for self-care and be incorporated into nursing practice as an educational resource, thus helping to promote self-care and the safety of elderly individuals after hospital discharge.

It is recommended that future research investigates the effectiveness of the booklet in clinical practice, as well as its applicability in different contexts and formats (e.g.. digital), to maximize its impact and reach).

## References

[1] World Health Organization (WHO) (2020). Report on progress in implementing the Global Strategy and Plan of Action on Ageing and Health 2016–2030: Information document.

[2] Ministério dos Direitos Humanos e da Cidadania (2023). Crescimento da população idosa traz desafios para a garantia de direitos.

[3] Pacífico  AACP, Feitosa  ESC, Parnaíba  ALS, Aquino  PL, Cavalcante  AA, Bezerra  TS (2022). Descriptive and temporal analysis of the mortality rate and average hospital stay due to burns and corrosion in the elderly in Brazil between 2010 and 2019. Rev Bras Cir Plást.

[4] Mrejen  M, Nunes  L, Giacomin  K (2023). Envelhecimento populacional e saúde dos idosos: O Brasil está preparado?.

[5] Anaya  M, Feinberg  G, Lopardo  T, Kheirbek  T (2024). Disparity in risk of readmission in adult burn patients: Analysis of a nationwide readmission database. J Surg Res.

[6] Bayuo  J, Wong  FKY, Agyei  FB (2020). “On the recovery journey:” An integrative review of the needs of burn patients from immediate pre-discharge to post-discharge period using the Omaha System. J Nurs Scholarsh.

[7] Mallmann  DG, Galindo Neto  NM, Sousa  J de C, Vasconcelos  EMR de (2015). Educação em saúde como principal alternativa para promover a saúde do idoso. Ciência & Saúde Coletiva.

[8] Silva  TM, Silvestrim  PR, Pereira  JTMO, Pimenta  RA (2023). Mobile applications for health education on the management of burns: Scoping review. Rev Bras Queimaduras.

[9] Feng  B, Liu  X, Shi  Y, Jiang  M, Chen  Y, Wang  Z (2025). Epidemiological features and temporal trends of burns and their subtypes, 1990–2021: Findings from the Global Burden of Disease Study 2021. Regen Repair Rehabil.

[10] Judd  K, Roberts  T, Guarrera  J (2025). Burn prevention outreach for older adults using county based meal delivery programs. J Burn Care Res.

[11] De Oliveira  MPS, dos Santos Alves  ME, Montezeli  JH, Milhorini  CR, Costa  DKC, Gastaldi  AB (2024). Validation of health educational technology for visitors of burned adults admitted to intensive care. Concilium.

[12] Fonseca  GP da, Souza  FT de, Rabito  LBF, Pereira  ND, Sanches  R de CN, Uema  RTB (2025). Tecnologia educativa para o atendimento inicial da equipe de enfermagem ao paciente adulto grande queimado. Texto Contexto Enferm.

[13] Goodman  C, Lambert  K (2023). Scoping review of the preferences of older adults for patient education materials. Patient Educ Couns.

[14] Echer  IC (2005). Elaboração de manuais de orientação para o cuidado em saúde. Revista Latino-Americana de Enfermagem.

[15] Ouzzani  M, Hammady  H, Fedorowicz  Z, Elmagarmid  A (2016). Rayyan—a web and mobile app for systematic reviews. Syst Rev.

[16] Alexandre  NMC, Coluci  MZO (2011). Validade de conteúdo nos processos de construção e adaptação de instrumentos de medidas. Cien Saude Colet.

[17] Melo  ESJ, Silva  MJN da, Silva  AP do N, Braga  HFGM, Oliveira  BSB de, Monteiro  FPM (2024). Criteria for selecting experts in the evaluation of educational technologies in nursing: An integrative review. Rev Rene.

[18] Polit  DF, Beck  CT (2019). Fundamentos de pesquisa em enfermagem: Avaliação de evidências para a prática da enfermagem.

[19] Leite  S de S, Áfio  ACE, Carvalho  LV de, Silva  JM da, Almeida  PC de, Pagliuca  LMF (2018). Construction and validation of an Educational Content Validation Instrument in Health. Rev Bras Enferm.

[20] Souza  ACC de, Moreira  TMM, Borges  JWP (2020). Development of an appearance validity instrument for educational technology in health. Rev Bras Enferm.

[21] Mattos  S, Moreira  T, Florêncio  R, Cestari  V (2021). Development and validation of an instrument to measure Self-Perceived Health in adults. Saude Debate.

[22] Damasceno  O (2025). “Cartilha Protegendo Sua Pele”. Mendeley Data V1.

[23] Alhussin  EM, Mohamed  SA, Hassan  AA, Al-Qudimat  AR, Doaib  AM, al jonidy  RM (2024). Patients’ satisfaction with the quality of nursing care: A cross-section study. Int J Afr Nurs Sci.

[24] Jawad  AM, Kadhum  M, Evans  J, Cubitt  JJ, Martin  N (2024). Recovery of functional independence following major burn: A systematic review. Burns.

[25] Pereira Lelis  JA, Espindula  AP (2020). Parametrização da limitação da amplitude de movimento articular (ADM) com os qualificadores da Classificação Internacional de Funcionalidade. Incapacidade e Saúde (CIF). Apae Cienc.

[26] Quintal  JS, Valduga  R, Suzuki  DRR (2023). Assessment of burn patients according to the International Classification of Functioning at hospital discharge: A cross-sectional study. Rev Bras Queimaduras.

[27] Buta  MR, Donelan  MB (2024). Evolution of burn care: past. present. and future. Clin Plast Surg.

[28] Gwyn-Jones  A, Afolabi  T, Bonney  S, Gurusinghe  D, Tridente  A, Mahambrey  T (2024). Major burns in adults: a practice review. Emerg Med J.

[29] Daronch  OT, Secanho  MS, Menezes  BF, Palhares  AA, Marcante  RFR (2023). Analysis of older patients hospitalized for burns in Brazil. Rev Bras Cir Plást.

[30] Rodrigues  MAC, Tanita  MT, Alfaro  AJY, Grion  CMC (2024). Patient care for burn victims in Brazil: A national survey. Burns.

[31] Moulin  LL, Dantas  DV, Dantas  RAN, Vasconcelos  EFL, Aiquoc  KM, Lima  KRB (2018). Perfil sociodemográfico e clínico de vítimas de queimaduras atendidas em um hospital de referêncial. Nursing.

[32] Su  YJ, Liang  SHY (2024). Unravelling the impact of prior depression and trauma-related cognitive processes on depression following trauma: A 2-year prospective study of burn survivors. Gen Hosp Psychiatry.

[33] Gowdar  TM, Handral  HV, Archana  G, Ramesha  KT (2025). Depression, anxiety and stress in post-burn patients and its association with self-esteem and social support: A cross-sectional study. J Psychiatry Spectrum.

[34] Carvalho  KM de, Figueiredo  M do LF, Galindo  NM, Sá  GG de M (2019). Construção e validação de cartilha para idoso acerca da higiene do sono. Revista Brasileira de Enfermagem.

[35] Rouzfarakh  M, Deldar  K, Froutan  R, Ahmadabadi  A, Mazlom  SR (2021). The effect of rehabilitation education through social media on the quality of life in burn patients: a randomized, controlled, clinical trial. BMC Med Inform Decis Mak.

[36] Martin  L, Andrews  S, Rea  S, Wood  F (2023). Motivating patients towards better recovery after burn: The development of a booklet to reframe perspectives. Burns.

[37] Coutinho  KB, Funchal  ACL (2022). Tecnologias educacionais em saúde relacionadas ao contexto do idoso com demência: Uma revisão integrativa. Rev Recien.

[38] Sun  KT, Shieh  TM, Hsia  SM, Ningrum  V, Lin  XY, Shih  YH (2021). Easy to read health education material improves oral health literacy of older adults in rural community-based care centers: a quasi-experimental study. Int J Environ Res Public Health.

[39] Kaminski-Ozturk  N, Reid  M (2024). A descriptive investigation of the nursing educator workforce in the United States. J Nurs Regul.

[40] Özdemir  A, Karadeniz  EY, Serin  EK (2024). Investigation of pre-discharge learning needs and affecting factors in individuals with burns. J Tissue Viability.

